# Blockchain adoption challenges in the healthcare sector: a waste management perspective

**DOI:** 10.1007/s12063-023-00413-9

**Published:** 2023-09-25

**Authors:** Sarthak Dhingra, Rakesh D. Raut, Vinay Surendra Yadav, Naoufel Cheikhrouhou, B. Koteswara Rao Naik

**Affiliations:** 1 Indian Institute of Management (IIM) Mumbai, Vihar Lake, Post-NITIE, Mumbai, Maharashtra 400087 India; 2 Operations and Quantitative Techniques, Indian Institute of Management Shillong, Meghalaya, 793018 India; 3 Geneva School of Business Administration, University of Applied Sciences Western Switzerland, HES-SO, Geneva, 1227 Switzerland; 4 IFM Business School, Geneva, Switzerland

**Keywords:** Blockchain, Healthcare, Reverse logistics, BWM, DEMATEL

## Abstract

The proposed study aims to identify the major challenges for blockchain adoption to manage reverse logistics activities of recyclable hospital waste in the Indian healthcare sector, in the COVID era. Fifteen challenges are identified through literature review and experts’ views and are prioritized and analyzed for cause-and-effect relationships using a hybrid approach combining Best–Worst Method (BWM) and Decision-Making Trial and Evaluation Laboratory (DEMATEL). A sensitivity analysis is performed to evaluate the results’ robustness. The results reveal that the Technological and Regulatory challenges category plays the most influential role consisting of Lack of Government Support and Policies, Lack of Strategic Planning, Lack of Knowledge and Qualified Expertise, Lack of Standards and Regulations, High Cost Involved, and Lack of Top Management Support are the most significant challenges affecting blockchain adoption. This study can support healthcare stakeholders, policymakers, government, and researchers in planning the strategic removal of the challenges to blockchain adoption in the Indian healthcare sector. The identification of the mutual interaction among the challenges will help healthcare decision makers address strategic questions of waste management from a holistic point of view. Since the work is achieved in the Indian healthcare context, generalization of the results must be carefully considered. The present study contributes significantly to discussing blockchain’s potential in healthcare waste management. The study’s findings can aid decision making process of managers, policymakers, and benefit researchers in this field.

**Supplementary Information:**

The online version contains supplementary material available at 10.1007/s12063-023-00413-9.

## Introduction

The healthcare sector provides the services, infrastructure, equipment, and medicines to support the well-being and health of a country’s citizens. Developing countries such as India generally face barriers to providing quality healthcare services (Zaprutko et al. 2020; Rodríguez et al. 2021). This concern has become more severe after the outbreak of the COVID-19 pandemic (Muduli et al. 2022). For the healthcare sector, waste generation is an essential concern due to the increased intake of medicines because of new drug development, life expectancy improvement, and quality of life (Hantoko et al 2021). Healthcare waste includes the solid and liquid waste generated from healthcare facilities and can cover a wide range of materials consisting of syringes, sampling needles, blood containers, pathological collections, chemical solutions, surgical devices, and radioactive materials. It is generated from laboratories, research centres, and healthcare facilities from patient’s examination, treatments, or immunization. Waste is defined as either hazardous or non- hazardous. Only 15% of medical waste is classified as hazardous waste that includes infectious, toxic, or radioactive waste and the remaining 85% of waste is general non-hazardous waste similar to that of a workplace or household, which includes packaging materials, food containers, and gloves worn to examine a non-infectious patient, and is where reductions or recyclability can be achieved (Xu and Yang 2022; Parida et al 2022). The pharmaceutical industry shows a growing trend due to the increasing consumption of medicines, population growth, and accessibility to medicines. Recently, the consumption of medicines by patients and waste generation by healthcare units increased due to the COVID-19 pandemic (Das et al. 2021; Džakula et al. 2022), mainly from the frequent use of consumables such as PPE kits, masks, gloves and disinfectants and contaminated waste, which has contributed to the increased severity of the problem. (De Campos et al. 2021; Buja et al. 2022; Rajan et al. 2022).

In the Indian context, the pharmaceutical sector is a highly competitive industry that aims to improve product visibility, while at the same time increase its reach to consumers. However, poor quality products in the disjointed distribution system have obstructed its sustainable practices (Narayana et al. 2019). Moreover, appropriate reverse logistics (RL) management of end-of-life medical devices, as well as recyclable waste and poor-quality items, is essential (Chan et al. 2012). As per the Executive Council of American Reverse Logistics, RL is the planning, controlling, and implementation of raw materials, inventory, finished goods, and related information from the origin point for value addition or appropriate disposal. RL is the upstream movement of medicines or medical equipment for value addition via transformation, or proper disposal in healthcare (Molano et al. 2022). Moreover, the strategic use of RL can help improve brand loyalty and customer satisfaction (Zhang et al 2011) and is a validated environmental strategy for waste management in developing countries (Daaboul et al. 2016; Chauhan and Singh 2021). Return of end-of-life products using RL allows to add value to the products transformed into new products.

Blockchain Technology (BT) can act as a solution for waste management and refurbishing medical devices whilst considering the operational, security, and regulatory requirements. BT is an open form of distributed ledger that helps record transactions between two parties and verify them permanently (Xu et al. 2022). The main objective of BT is to create a peer-to-peer network and a distributed database that counts on solid cryptographic support (Tian et al. 2022) and facilitates transparent and secure information exchange in the supply chain. Blockchain is of mainly four types- public, private, consortium and hybrid. Public blockchain, also called permissionless blockchain, is a decentralized blockchain with no organization or individual controlling it. Anyone can access the network and participate in its core activities showing its self-governed and decentralized nature. Its advantages include easy accessibility, immutability, as transactions are recorded and can’t be altered, and high security, due to the presence of many nodes in the network to be hacked. Its disadvantages include low throughput and high consumption of energy. Its applications include Bitcoin, Ethereum and Litecoin (Bamakan et al. 2022). A private blockchain, also called permissioned blockchain, has a single entity or operator who decides who can access the network as well as who can view and create data on blockchain. A private blockchain requires users access verification, which is not in the case of public blockchain. It is either centralized or partially decentralized. Private blockchains may have an advantage of greater speed of processing transactions as the users are limited to a particular organization who need to achieve consensus to validate transactions. Despite the advantages of faster, efficient, and trusted systems, private blockchains present some disadvantages too; They are less secure and can be edited by the operator. Its applications include Hyperledger or Ripple (Mogale et al. 2022a, b). To overcome the disadvantages of public and private blockchain, consortium and hybrid blockchains come into the picture. Consortium blockchain is a permissioned federated blockchain which is used when different organizations come together to share data or make transactions. So, its decentralized nature increases as compared to private blockchains and so the security. Hybrid blockchain is a combination of permissioned and permissionless blockchain and has a balance of both control and freedom. It has combined benefits of both public and private blockchain and can execute permissioned as well as permissionless features depending upon the need of the situation (Hrouga et al. 2022). Controlling authorities can keep the transactions open for public or make it transparent. Hybrid blockchains are the most suitable for healthcare sector as data sharing may be required within a hospital or between different healthcare partners depending on the situation.

BT practices could help develop environmentally sound solutions by reducing natural resources’ wastage through reusing and recycling during the reverse flows (Morgan et al. 2016; Xu and He 2022). BT is an appropriate solution for the creation of an efficient management system that can manage recalled goods, their placement in inventory, recycling, disposal, repair, and resale (Saberi et al. 2019). The increased transparency introduced by BT supports inventory management, raw material quality control, and reduce product recalls. It minimises delays in data collection, leading to fast and effective decisions, and thus reduces the effort and time required to recall a product. Traditional paperwork is time consuming, prone to human error, and involves third parties. Smart contracts use BT to resolve these concerns by eliminating intermediaries, digitalising the information flows, which in turn increases the environmental friendliness and reduces time (Rana et al. 2022). Smart contracts are programs stored on blockchain that work on fulfilling predetermined conditions. Blockchain features such as decentralization, immutability, distributed ledger, and consensus mechanisms help to take quick and unbiased decisions and provide enhanced security (Toufaily et al. 2021). Furthermore, according to Bamakan et al. (2022), BT outperforms traditional techniques in managing quicker transactions. In their study, they looked at how Walmart utilized BT to track the source of mangoes in only a few seconds, compared to seven days using traditional methods. BT can be used to manage and recycle returned goods to reduce environmental damage and facilitate better planning for recycling organizations (Queiroz and Wamba, 2019). BT ensures the authentication of products as counterfeit products negatively impact the manufacturers and service providers and affect consumer trust. This reduces time, materials, and energy waste and improves consumers’ trust. Since a vast amount of data is generated and shared every day in the healthcare industry, data security becomes a concern across healthcare supply chains. To cope with this problem, BT helps provide the required security and privacy to avoid fraud and integrate forward and reverse flows (Khan et al. 2021).

BT application in forwarding supply chains has been researched well but its application in RL is still unexplored (Malolan et al 2020). Moreover, the literature focuses on the techniques to manage the waste management issues rather than understanding the challenges for BT adoption to tackle waste management problems (Bamakan et al. 2022). Despite several benefits of the technology, there are a few challenges in BT’s implementation in the Indian healthcare sector for managing recyclable hospital waste. These challenges include the constraints that should be removed for the smooth adoption of BT in the healthcare system for efficient waste management. Therefore, there is a strong need to revisit these adoption challenges in the healthcare environment, particularly in the Indian context which leads us to the following research question: *What are the challenges to BT adoption in the Indian healthcare sector for managing recyclable hospital waste?* Knowing what obstacles exist allows for the creation of a strategy to overcome them, but not all challenges are equal in importance. Some of them are given greater weight, impact, and consideration because they are more significant, and even these challenges can interact with one another. It is crucial to prioritize these challenges according to their relative importance and, with the advice of experts, determine whether there are any contextual linkages between them. Consequently: *What are the most dominant challenges for BT adoption in the Indian healthcare sector to manage recyclable hospital waste, and what contextual relationships do they possess?* By answering this question, healthcare stakeholders will gain an understanding of the most important challenges among them as well as their relationships with one another.

In general, this paper clarifies how blockchain might be used to manage recyclable hospital waste in the context of Indian healthcare. This study is original from the perspective of Indian healthcare waste management and will help managers, policymakers, and researchers who are involved in this field make decisions. Its analysis, interpretation, and results are achieved using a combined MCDM approach that includes the Best Worst Method for prioritizing challenges and DEMATEL for identifying contextual relationships. This research will contribute to a better understanding of the challenges themselves and how they affect policy and decision-making. The remaining part of the paper is structured as follows: Section 2 presents the literature review conducted for the study. Section 3 discusses the detailed research methodology adopted in this work. The results and analysis part are shown in Section 4, whereas discussion of the results is presented in Section 5. Section 6 provides the managerial implications of the conducted work. Finally, conclusion and the future work directions are presented in Section 7.

## Literature review

The research is intended to identify the challenges related to BT adoption in the Indian healthcare sector and analyse their interrelationships. The bibliographic databases Web of Science, Scopus, and Science Direct, are searched for relevant articles. Feasible combinations of the keywords search strings such as (‘blockchain’, ‘healthcare*’, and ‘*challenges’)* and (‘waste management*’, ‘healthcare*’, and ‘*challenges’)* were used to find relevant articles for this study. ‘Healthcare*’ was alternatively used with *‘smart healthcare’, ‘medical’*, *‘e-health’*, *‘pharmaceutical’*, *‘healthcare service’, ‘healthcare management’* and *‘healthcare system’*. In the same way, ‘waste management*’ was alternatively used with ‘reverse logistics’ to avoid false negatives and complete the search criteria.

### Nexus of BT in waste management, reverse logistics, and healthcare

Narayana et al. (2019) studied the complexities of Indian pharmaceutical supply chains and proposed changes to enhance sustainable performance. The authors explained how improvements in RL attributes can reduce wastage using a system dynamics model. Malolan et al. (2020) highlighted that there is sufficient research in forward supply chains but research on BT application in RL is still lacking. Douladiris et al. (2020) proposed a BT-based framework for RL operations to refurbish medical devices considering the security, regulatory and operational needs. In the Brazilian context, Vieira et al. (2020) analysed the RL challenges in e-waste. The study revealed that ‘regulations and policy’ and ‘market and social’ are the two main challenges that hinder RL adoption. Massaro (2021) presented views about the digital transformation of the healthcare industry using BT since the latter can help to speed up the process of digital transformation by solving medical practice challenges. Bekrar et al. (2021) analysed the relationship between BT, RL, and transportation. They proved that digitalising the RL and transportation activities with BT need changes in beliefs, policies, and practices. Shih et al. (2021) proposed a BT-based system to prevent fraudulent activities in the return and exchange of goods using smart contracts. It helps to reduce manpower for monitoring purposes and the import of counterfeit goods. Hrouga et al. (2022) proposed digitizing reverse supply chains based on IoT-blockchain for treating hazardous waste. The authors suggested conceptual models for open and closed-loop supply chains and verified them with a case study. Ghadge et al. (2022) established the link between industry 4.0 technologies and green supply chain management an highlighted that green purchasing and reverse logistics are highly influenced by industry 4.0 technologies and also impact the green SC performance. Omar et al. (2022) planned a BT-based result using smart contracts to manage the operations of PPE supply chains. The solution ensures trust, security, and transparency in communication among the stakeholders. Bamakan et al. (2022) presented the contributions of BT in solving environmental issues. The authors discussed BT’s potential to meet healthcare waste management needs and proposed BT-based solutions for managing hospitals waste. They stated that the focus of literature has been in understanding and developing waste management techniques rather than investigating BT adoption challenges for tackling waste management issues.

### Applications of BT in healthcare waste management

Dogo et al. (2019) argued that the security and transparency aspects of BT for sewage treatment can improve system efficiency and reduce operating costs. Sløgedal and Starling (2020) used BT as a tool for identification and security improvement in the context of tracking medical waste packages. Pournader et al. (2020) proposed that BT can help establish trust, traceability, better trade, and transparency within the system for waste transportation purposes. Koh et al. (2020) proposed a BT-based intelligent waste transportation system within the hospital for waste separation and transportation ensuring reduced traffic. Liu et al. (2021) attempted classification of different types of plastic waste using BT to accelerate its recycling. Gopalakrishnan et al. (2021) shared information regarding solid plastic waste identification and collection among different organizations using BT for recycling purposes.

### Challenges for BT adoption with respect to recyclable waste management

Yaqoob et al. (2019) mentioned the lack of clear regulations, technical issues, and resistance to change as substantial challenges for BT’s implementation in the healthcare scenario. Kassab et al. (2019) studied BT’s status in healthcare and reported financial constraints, top management support, government support, and infrastructure requirements as significant challenges. Sharma and Joshi (2021) analysed BT’s implementation challenges concerning the Indian healthcare sector and proposed regulation issues, interoperability and security as common issues faced. Spanò et al. (2021) explored how BT can add value to the healthcare sector. Lack of trust, high cost involved, and unclear vision and mission are the challenges faced in technology’s adoption in the healthcare sector. Karakas et al. (2021) studied BT adoption status in logistics and supply chains. The authors pointed out that lack of strategic planning, top management support, and lack of financial support are significant challenges to BT adoption. Erol et al. (2022) examined the impact of the challenges to circular economy adoption through BT. The authors suggested that low trust, lack of collaboration among stakeholders, and technical challenges are the ones that need to be overcome for BT’s implementation. Dominic et al. (2021) studied the challenges of adopting RL in manufacturing. The authors mentioned lack of infrastructure and low technical expertise as highly prioritised challenges. Lima et al. (2022) presented a systematic literature review on pharmaceutical RL operations. The authors proposed lack of environmental awareness, lack of governmental policies, and lack of regulations as the main issues of RL adoption.

### Tools and techniques used

Kargar et al. (2020) proposed a network design model of RL for managing medical waste during COVID. The authors minimized the cost and risk of handling infectious waste through multi-objective goal programming. Rana et al. (2022) explored BT adoption challenges using ISM-MICMAC analysis within the Indian public sector. Mangla et al. (2021) analysed challenges to food safety in emerging economies. The authors used the BWM-DEMATEL integrated technique to prioritize the challenges and establish causal relationships. Mahmud et al. (2021) analysed the collaboration barriers in supply chains in SMEs with the help of grey DEMATEL and fuzzy BWM. The authors compared the results from two different MCDM techniques. Kaswan et al. (2021) explored green six-sigma adoption challenges for assessing sustainability in manufacturing. The authors analysed the results using the integrated technique of BWM and DEMATEL. Bai et al. (2021) evaluated the service vendor platform selection based on BT. The authors used BWM, DEMATEL, and social network theory for the evaluation. Kannan (2021) examined sustainable procurement drivers in a Danish supply chain context. BWM was used to prioritize the drivers, and an interrelationship analysis was done using DEMATEL. Dominic et al. (2021) studied the challenges of adopting RL in manufacturing. The authors used DEMATEL and EDAS for prioritization and interrelationship analysis.

### Motivation for the study and research gaps

As there is a rapid evolution of blockchain-related concepts and implements, the exploration of applications of BT into RL has become a research area of interest (Morgan et al. 2018). Samson (2020) emphasizes on the need to integrate emerging technologies such as BT to help companies withstand the shocks due to pandemics or crises. He suggests the necessity of restructuring the reverse logistics system by incorporating BT. A significant amount of work has been published regarding BT application in forwarding supply chains. However, BT application in RL is still unexplored (Lagorio et al. 2020; Malolan et al. 2020), which constitutes a first gap. Moreover, the focus of the literature is on the use of suitable tools and techniques to handle the waste management issue (Bamakan et al. 2022) rather than understanding the challenges and the issues from a systematic point of view. Therefore, there is a need for empirical studies to find how BT incorporation can improve waste management operations (Kamble et al. 2020; Nguyen et al. 2020). This paper addresses the identified research gaps in identifying the challenges in the adoption of BT in the management of hospitals waste in a reverse logistics perspective.

## Research methodology

**Fig. 1:**
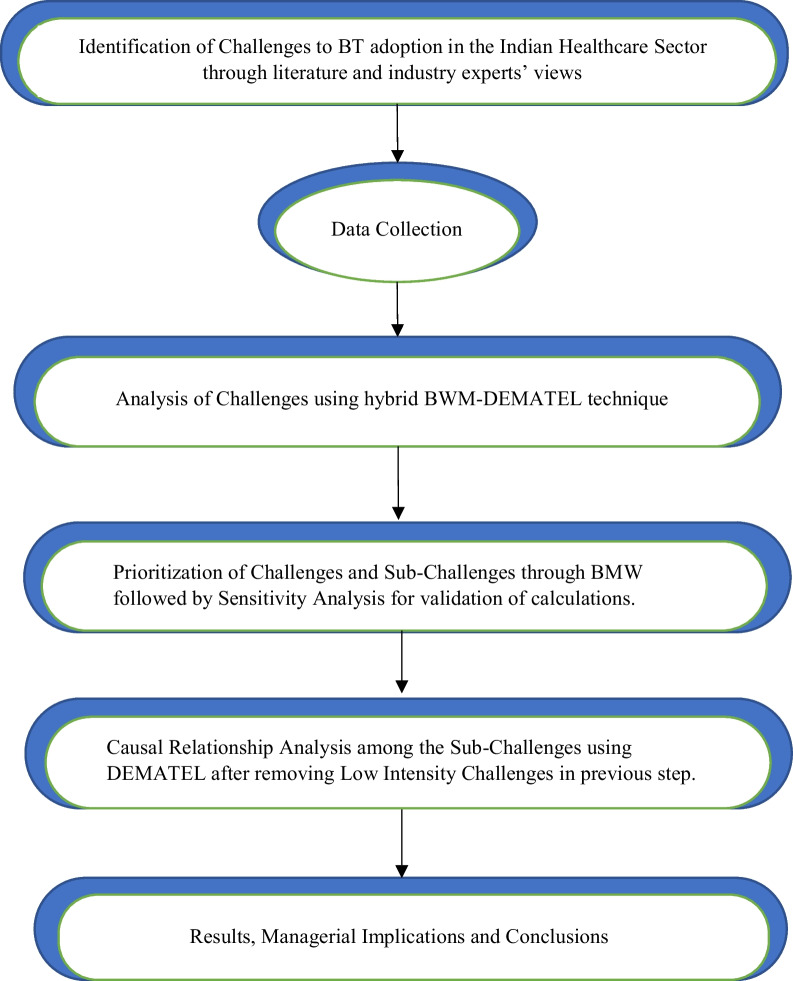
Research methodology

The Multi-Criteria Decision Making (MCDM) problem treated in this study must identify the most important challenges as well as analyse the interdependencies among them based on different criteria and objectives. This study proposes a hybrid MCDM approach, BWM-DEMATEL, to achieve the research objectives according to the methodology shown in Fig. 1. The first step is the identification of the challenges through a literature search and industry experts’ survey, followed by the data collection process and further analysis using a combined BWM-DEMATEL technique. Both the BWM and DEMATEL have been extensively used individually in the literature, however the combined approach of BWM-DEMATEL used for the Indian healthcare context in this study contributes to the technical novelty of the work. The two techniques play different roles; BWM prioritizes the importance of the challenges, and DEMATEL evaluates the causal relationships between them. The hybrid approach proposed is suitable for this study as the techniques are complementary and offset each other’s shortcomings (Govindan et al. 2020; Fartaj et al. 2020). BWM is an effective decision-making tool for answering practical problems. It ranks the challenges using feedback from experts (Yadav et al. 2018). Its unique advantages that make it preferable over other MCDM methods include: 1) BWM requires a smaller number of experts to produce effective decisions (Tarei et al. 2021), which makes it desirable for this study, given that only a small number of blockchain experts are available; 2) Fewer pairwise comparisons are involved in this method as compared to other MCDM methods such as AHP, F-AHP, Grey DEMATEL, or DEMATEL (Paul et al. 2021); 3) Requires less computational time and effort compared to other MCDM methods such as TOPSIS, DEMATEL, and ANP (Moktadir et al. 2019); 4) Reduced comparison complexity due to the use of integer scale of 1–9 (Moktadir et al. 2019). DEMATEL is based on graph theory and permits a visual understanding of influencers as well as the cause and effect relationships between the challenges. It effectively analyses both direct and indirect mutual influences and also measures criteria weights (Rajput and Singh 2019; Bumblauskas et al. 2020).

### Best worst method (BWM)

BWM is an MCDM technique that uses pairwise comparison among the challenges to find their optimal weights (Rezaei 2015). In this method, the reliability and consistency of the results are checked with the Consistency Ratio (CR). The implementation of the procedure is as follows:

Stage 1: Challenges to BT adoption in the Indian healthcare sector for managing RL issue of recyclable waste are identified with the help of a literature review and experts’ views.

Stage 2: The most important or “best” criteria and least important or “worst” criteria are chosen among the challenges through expert views and pairwise comparisons.

Stage 3: After selecting the best and worst criteria, choosing the best challenge concerning other challenges is decided using a rating scale of 1–9. Best-to-Others vector is ${v}_{B}=( {v}_{b1, }{v}_{b2,\dots \dots \dots .. , }{v}_{bn })$, where ${v}_{Bj}$ is the preference of best challenge B over criteria j and ${v}_{BB}=1$.

Stage 4: Further, preference for other challenges concerning the worst challenge is determined using the rating scale of 1–9. The vector Others-to-Worst (OW) is ${v}_{w}={\left({v}_{1w}, {v}_{2w},\dots \dots \dots , {v}_{nw}\right)}^{T}$, where ${v}_{jw}$ denotes the preference of challenge j over worst criteria w and ${v}_{ww}=1$.

Stage 5: The optimal weights of the challenges are determined. They are calculated such that the maximum absolute difference $\{ {|w}_{B}-{v}_{Bj}{w}_{j}\left|, {|w}_{j}-{v}_{jw}{w}_{w}\right|\}$ for all j is minimized.

Min δ

Subject to,

$${|w}_{B}-{v}_{Bj}{w}_{j}|\le \delta \forall j$$

$${|w}_{j}-{v}_{jw}{w}_{w}|\le \delta \forall j$$

1 $$\sum {w}_{j}=1$$

$${w}_{j}\ge 0 \forall j$$

The equations are solved, and the optimal weights are computed while minimising the value of δ. A value of δ closer to 0 shows high consistency of the results.

### Decision-making trial and evaluation laboratory (DEMATEL)

DEMATEL is used to analyse interrelationships among the factors using diagraphs or matrices of a system. It helps to determine the strength and dependency of the relationships among the factors. The stages involved in this technique are as follows:

Stage 1: An initial direct relation matrix $E=\{{e}_{ij}\}$ is constructed from a set of factors $\mathrm{f }= \left\{\mathrm{fi}=\mathrm{1,2},\dots .,\mathrm{n}\right\}$ by decision-makers using a linguistic scale of five levels varying from “No Influence” to “Very High Influence”.

Stage 2: Formation of normalised direct-relation matrix (N) with the help of (2) and (3)

2 $$N=s.E$$

3 $$\mathrm{s }= 1/ ({max}_{1\le i\le n} \sum\nolimits_{j=1}^{n}{e}_{ij}),\mathrm{ i},\mathrm{ j }=\mathrm{ 1,2},\dots ,\mathrm{n}$$

Stage 3: Formation of Total relation matrix (M) using (4) –

4 $$\mathrm{M }=\mathrm{ N }{\left(\mathrm{I}-\mathrm{N}\right)}^{-1}$$

The total relation matrix represents the final relationship among the factors after continuous iterations of direct and indirect relations.

Stage 4: Calculation of row (D) and column (R) sums from the total relation matrix (M).

Stage 5: Evaluation of causal/effect diagram using (D + R) and (D-R) values.

Multi-speciality hospitals in India are known for providing quality healthcare. They recognise the importance of incorporating digital technologies for better maintenance and sharing of healthcare records, transparent clinical results, and traceability improvement in supply chains. For achieving technological advantage and making healthcare services better, these hospitals are keen to adopt BT. BT-based healthcare start-ups are also contacted for the data collection process in addition to managers, CEOs, medical staffs, engineers of healthcare units, and doctors. This can be referred in Table A8 of the supplementary material**.**

## Results

This section presents the results and analysis conducted in the study. Firstly, the challenges and sub-challenges weights are estimated by BWM. The calculations of BWM are then checked with the aid of sensitivity analysis for validation. The sub-challenges are further subjected to DEMATEL to check their cause-and-effect relationships.

After a first round of consultation with experts, these sub-challenges are resubmitted to them for their classification into respective categories based on their perspectives. The consensus amongst the experts is observed to classify the identified barriers into four categories as shown in Table 1. A description of the challenge with respect to its category among the four categories: Organizational and Management Challenges (I), Supply Chain and Collaboration Challenges (II), Economic and Environmental Challenges (III), Technological and Regulatory Challenges (IV) and the supporting references are provided.

**Table 1:** Identified challenges for BT adoption in Indian healthcare sector for managing hospital recyclable waste

**Category**	**Sub-Challenges**	**Description**	**Source**
Organizational and Management Challenges (I)	Resistance to Change (C1)	The culture of healthcare organizations to work in a traditional way makes the technology’s acceptance for recyclable waste management difficult	Kassab et al. (2019); Yaqoob et al. (2019), Rana et al. (2022), Govindan et al. (2022)
	Unclear Mission and Vision regarding Waste Management (C2)	Unclear goals, insufficient commitment, and preparation for the technology’s use for providing sustainable waste management solutions exits in Indian healthcare sector	Sharma and Joshi (2021), Spanò et al. (2021), Govindan et al. (2022)
	Lack of Strategic Planning for Sustainable Operations (C3)	Today, healthcare companies lack clear strategies and planning regarding the blockchain use for hospital recyclable waste management	Dominic et al. (2021), Karakas et al. (2021), Toufaily et al. (2021)
	Lack of Top Management Support (C4)	Hospitals’ top management’s unclear strategies and insufficient monetary support for the technology’s usage prevails in Indian medical waste management	Yaqoob et al. (2019), Karakas et al. (2021), Govindan et al. (2022)
Supply Chain and Collaboration Challenges (II)	Privacy Issues (C5)	The fear of compromise of confidential data as the technology is nascent, with very few implementations in the Indian healthcare scenario. The organizations sometimes have intellectual property concerns regarding waste produced and waste management practices followed	Shi et al. (2017), Spanò et al. (2021), Erol et al. (2022), Govindan et al. (2022), Yadav and Kumar (2023)
	Lack of Trust among Supply Chain Partners (C6)	The healthcare stakeholders lack confidence in each other, doubt information sharing on a common platform due to alignment of conflicting strategies throughout the supply chain	Yaqoob et al. (2019), Rana et al. (2022), Erol et al. (2022), Govindan et al. (2022), Yadav and Kumar (2023)
	High-Cost of Technology and Environmentally Sustainable Operations (C7)	Different costs involved are regarding network maintenance, installation, development, and hiring of technical experts as well as managing BT enabled recyclable hospital waste operations	Chauhan et al. (2018), Sharma and Joshi (2021), Spanò et al. (2021), Govindan et al. (2022), Yadav and Kumar (2023)
Economic and Environmental Challenges (III)	Lack of Financial Support (C8)	Lack of monetary assistance from the government’s and the management's side is an obstacle for using blockchain for cost-effective medical waste management	Chauhan et al. (2018), Yaqoob et al. (2019), Karakas et al. (2021), Govindan et al. (2022)
	Lack of Environmental Awareness and Green Practices (C9)	Limited environmental knowledge and inadequate sustainability practices in BT and RL reduce faith	Liu et al. (2013), Kurdve et al. (2015), Dominic et al. (2021), Lima et al. (2022)
	Uncertain Return on Investment (C10)	Risk is involved to replace the traditional working with new technology with no proven successful implementations for recyclable waste management	Expert’s opinion
Technological and Regulatory Challenges (IV)	Lack of Government Policies for BT enabled Waste Management (C11)	Government policies regarding blockchain working and monetary support are essential for blockchain enabled healthcare waste recycling units to dwell	Yaqoob et al. (2019), Torkayesh et al. (2021), Govindan et al. (2022), Lima et al. (2022), Yadav and Kumar (2023)
	Lack of Knowledge and Qualified Expertise (C12)	Inadequate knowledge about the technology and RL benefits and lack of blockchain developers in India make the healthcare organizations doubt its implementation for recyclable waste management	Dominic et al. (2021), Govindan et al. (2022), Yadav and Kumar (2023)
	Technical Challenges to BT controlled Recyclable Waste Management (C13)	Technical issues like interoperability, low scalability, throughput, latency, and storage issues are generally faced while the technology’s adoption for managing recyclable waste	Sharma and Joshi (2021), Erol et al. (2022), Govindan et al. (2022), Yadav and Kumar (2023)
	Lack of Proper Infrastructure (C14)	Inappropriate technical and physical infrastructure for blockchain adoption for hospital waste management needs attention	Dominic et al. (2021), Govindan et al. (2022), Yadav and Kumar (2023)
	Lack of Standards and Regulations regarding Waste Recycling (C15)	There are no standard protocols regarding the adoption and use of the technology for recyclable waste management in India. Different healthcare organizations follow different regulations and standards without a common regulatory framework. Also, the government, hospital and stakeholder organization laws shall also comply with BT standards and regulations	Sharma and Joshi (2021), Lima et al. (2022), Govindan et al. (2022), Yadav and Kumar (2023)

### Weights estimation using BWM

The best and worst challenges are first identified with the help of professionals. Table 2 shows the selected “best” and “worst” challenges as recognized by experts.

**Table 2:** Recognition of “Best” and “Worst” challenge and sub-challenge by healthcare professionals

**Challenges and Sub-Challenges**	**Identified as “Best” by Professionals**	**Identified as “Worst” by Professionals**
**Category Challenges**		
Organizational and Management	4,6,8,	2,5,7,15
Supply Chain and Collaboration	1,13,11	3,4,6,8,9,12,14
Economic and Environmental	14	10,13
Technological and Regulatory	2,3,5,7,9,10, 12, 15	1, 11
**Sub-challenges of Organizational and Management Category**		
Resistance to Change	1	2,3,5,8,9,11,14
Unclear Mission and Vision	2,4,8,11,14	1,4,6,13
Lack of Strategic Planning	3,5,6,7,9,10, 12	15
Lack of Top Management Support	13,15	7,10,12
**Sub-challenges of Supply Chain and Collaboration Category**		
Lack of Trust	9, 11,13,14	1,3,4,5,7,10,12
Lack of Collaboration among Supply Chain Partners	1,3,7	2,6,8,15
High Cost Involved	2,4,5,6,8,10,12, 15	9,11,13,14
**Sub-challenges of Economic and Environmental Category**		
Lack of Financial Support	1,3,4,5,8,10,12,13	7,11,14
Lack of Environmental Awareness	2,6,7,15	1,4,9,10
Uncertain Return on Investment	9,11,14	2,3,5,6,8,12,13,15
**Sub-challenges of Technological and Regulatory Category**		
Lack of Government Support and Policies	1,3,4,7,9,11	12,14
Lack of Knowledge and Qualified Expertise	2,5,8	9,13,15
Technical Challenges	6,10	8,11
Lack of Proper Infrastructure	12,15	3,5,6,7,10
Lack of Standards and Regulations	13,14	1,2,4

The “Best-to-Others” (BO) matrix and the “Others-to-Worst” (OW) matrix present the pairwise comparison among the challenges and sub-challenges. The comparison is made using a scale of 1–9, where 1 represents the equal importance and 9 represents the extreme importance. Table 3 shows the BO and OW matrix with evaluation scores by experts.

**Table 3:** ‘Best-to-Others’ and ‘Others-to-Worst’ matrix for challenges

**Best to Others (BO)**	**Organizational and Management Challenges**	**Supply Chain and Collaboration Challenges**	**Economic and Environmental Challenges**	**Technological and Regulatory Challenges**
Technological and Regulatory Challenges	2	4	6	1
**Others to worst (OW)**		**Worst Challenge (Economic and Environmental Challenges)**		
Organizational and Management Challenges		3		
Supply Chain and Collaboration Challenges		2		
Economic and Environmental Challenges		1		
Technological and Regulatory Challenges		6		

The preference scores given by experts are used to compute the local weights of challenges and sub-challenges. Then, local weights are multiplied by main category weights to get the global weights. Prioritization of the challenges is decided based on the global weights, as shown in Table 4. The reliability and consistency of the data can be checked with the help of the Consistency Ratio (CR) whose value should be less than 0.1.

**Table 4:** Prioritization of challenges based on BWM

**Category**	**Categorical Weights**	**Challenges**	**Local Weights**	**Global Weights**	**Ranks**
Organizational and Management Challenges (I)	0.27	Resistance to Change (C1)	0.09	0.0243	12
		Unclear Vision and Mission regarding Waste Management (C2)	0.14	0.0378	10
		Lack of Strategic Planning for Sustainable Operations (C3)	0.50	0.1350	2
		Lack of Top Management Support (C4)	0.27	0.0729	6
Supply Chain and Collaboration Challenges (II)	0.14	Privacy Issues (C5)	0.17	0.0238	13
		Lack of Trust among Supply Chain Partners (C6)	0.29	0.0406	9
		High Cost of Technology and Environmentally Sustainable Operations (C7)	0.54	0.0756	5
Economic and Environmental Challenges (III)	0.08	Lack of Financial Support (C8)	0.62	0.0496	8
		Lack of Environmental Awareness and Green Practices (C9)	0.25	0.0200	14
		Uncertain Return on Investment (C10)	0.13	0.0104	15
Technological and Regulatory Challenges (IV)	0.51	Lack of Government Policies for BT enabled Waste Management (C11)	0.41	0.2091	1
		Lack of Knowledge and Qualified Expertise (C12)	0.24	0.1224	3
		Technical Challenges to BT controlled Recyclable Waste Management (C13)	0.12	0.0612	7
		Lack of Proper Infrastructure (C14)	0.07	0.0357	11
		Lack of Standards and Regulations regarding Waste Recycling (C15)	0.16	0.0816	4

**Fig. 2:**
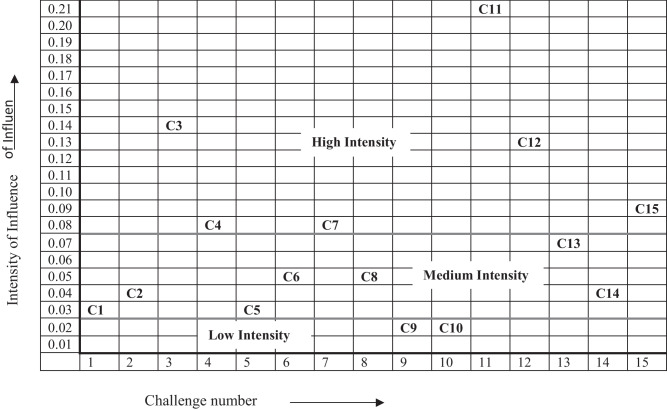
Categorization of sub-challenges based on their intensity of influence

Figure 2 shows the categorization of sub-challenges centred on their intensity of influence. Based on the experts’ views after the prioritization, all the sub-challenges are grouped into three categories because of their respective weights i.e., low, medium, and high intensity. It can be noticed that (C3), (C4), (C7), (C11), (C12) and (C15) are among the sub-challenges with the highest intensity of influence.

### Sensitivity analysis

After prioritizing challenges to BT adoption in the Indian healthcare sector for managing recyclable hospital waste, the robustness and accuracy of the calculations by BWM are checked. Sensitivity analysis is a tool that helps in achieving the same. This study has been performed by varying the weight of the highest weighted Technological and Regulatory Challenges category from 0.1 to 0.9, as shown in Table 5. As the category's weight is varied, other categorical weights are adjusted so that their sum equals one, while challenges’ local weights remain constant. The variation in the ranks of challenges can be seen in Table 6.

**Table 5:** Variation in weight of category (IV) from 0.1 to 0.9

**Selected Category**	**Normal (0.51)**	**Preference Weights for Various Categories**								
		**0.1**	**0.2**	**0.3**	**0.4**	**0.5**	**0.6**	**0.7**	**0.8**	**0.9**
**I**	0.27	0.50	0.44	0.38	0.32	0.26	0.20	0.14	0.08	0.02
**II**	0.14	0.26	0.23	0.20	0.17	0.14	0.11	0.08	0.05	0.02
**III**	0.08	0.14	0.13	0.12	0.11	0.10	0.09	0.08	0.07	0.06
**IV**	0.51	0.10	0.20	0.30	0.40	0.50	0.60	0.70	0.80	0.90
**Total**	1	1	1	1	1	1	1	1	1	1

**Table 6:** Variation in ranks of the challenges obtained with sensitivity analysis

**Sub-challenges**	**Normal Ranks (0.51)**	**Ranks of Sub-Challenges according to Variable Weights**								
		**0.1**	**0.2**	**0.3**	**0.4**	**0.5**	**0.6**	**0.7**	**0.8**	**0.9**
**C1**	12	7	9	11	12	14	14	14	15	15
**C2**	10	6	7	8	10	10	11	12	12	14
**C3**	2	1	1	1	2	2	3	5	7	9
**C4**	6	3	3	4	5	6	8	9	9	12
**C5**	13	8	10	12	11	13	13	13	14	13
**C6**	9	5	6	7	8	9	10	10	11	11
**C7**	5	2	2	3	4	5	6	8	8	8
**C8**	8	4	5	5	6	7	7	6	6	6
**C9**	14	10	11	13	14	12	12	11	10	7
**C10**	15	12	14	15	15	15	15	15	13	10
**C11**	1	9	4	2	1	1	1	1	1	1
**C12**	3	11	8	6	3	3	2	2	2	2
**C13**	7	14	13	10	9	8	5	4	4	4
**C14**	11	15	15	14	13	11	9	7	5	5
**C15**	4	13	12	9	7	4	4	3	3	3

With the help of sensitivity analysis, it is evident that the high-intensity sub-challenges (C3, C4, C7, C11, C12, and C15) generally remain in the top six sub-challenges as the weight of the highest weighted category is varied from 0.1 to 0.9. Lack of Government Support and Policies for BT enabled Waste Management (C11), Lack of Strategic Planning for Sustainable Operations (C3), and Lack of Knowledge and Qualified Expertise (C12) are the top three sub-challenges in most cases. The low-intensity sub-challenges of Lack of Environmental Awareness and Green Practices (C9) and Uncertain Return on Investment (C10) are mostly the least placed ones. Table 6 shows that the medium intensity sub-challenges generally rank between high intensity and low-intensity sub-challenges in the sensitivity analysis. On consultation with industrial and academic experts, the low intensity sub-challenges C9 and C10 are dropped after their approval as they don’t possess direct influence and are influenced by other sub-challenges. Further, DEMATEL analysis is carried out with the left thirteen sub-challenges to determine their cause-and-effect relationships.

**Table 7:** Total relation and direct–indirect influence matrix

	C1	C2	C3	C4	C5	C6	C7	C8	C11	C12	C13	C14	C15	**D (sum of row)**	**D + R**	**D-R**	**Cause-effect group**
C1	0.32	0.43	0.46	0.32	0.41	0.38	0.45	0.51	0.52	0.46	0.45	0.49	0.53	5.73	9.82	1.64	cause
C2	0.34	0.30	0.37	0.28	0.35	0.33	0.38	0.44	0.45	0.38	0.41	0.43	0.45	4.91	9.00	0.46	cause
C3	0.36	0.38	0.33	0.31	0.36	0.32	0.43	0.47	0.47	0.39	0.41	0.47	0.47	5.17	9.26	0.49	cause
C4	0.29	0.31	0.34	0.20	0.28	0.30	0.30	0.39	0.41	0.33	0.34	0.35	0.35	4.19	8.28	0.66	cause
C5	0.32	0.34	0.31	0.27	0.27	0.31	0.34	0.39	0.41	0.35	0.39	0.38	0.39	4.46	8.55	0.09	cause
C6	0.29	0.27	0.28	0.25	0.27	0.21	0.28	0.34	0.33	0.30	0.30	0.30	0.34	3.76	7.85	-0.28	effect
C7	0.33	0.38	0.39	0.29	0.36	0.34	0.33	0.42	0.47	0.44	0.41	0.43	0.46	5.06	9.15	0.28	cause
C8	0.33	0.34	0.39	0.28	0.36	0.32	0.38	0.36	0.48	0.42	0.41	0.43	0.45	4.96	9.05	-0.44	effect
C11	0.30	0.31	0.36	0.28	0.35	0.30	0.38	0.44	0.36	0.41	0.41	0.41	0.39	4.70	8.79	-0.93	effect
C12	0.30	0.33	0.36	0.27	0.34	0.31	0.40	0.42	0.44	0.32	0.38	0.43	0.43	4.73	8.82	-0.24	effect
C13	0.26	0.33	0.29	0.23	0.34	0.28	0.33	0.37	0.41	0.34	0.28	0.37	0.34	4.17	8.26	-0.80	effect
C14	0.32	0.34	0.37	0.27	0.33	0.30	0.34	0.40	0.43	0.38	0.37	0.34	0.45	4.64	8.73	-0.65	effect
C15	0.34	0.39	0.42	0.28	0.35	0.35	0.41	0.45	0.45	0.44	0.41	0.47	0.38	5.14	9.23	-0.27	effect
sum of column R	4.09	4.45	4.68	3.53	4.36	4.04	4.78	5.40	5.63	4.98	4.97	5.29	5.42	61.63			
													α	0.36			

### DEMATEL analysis

**Fig. 3:**
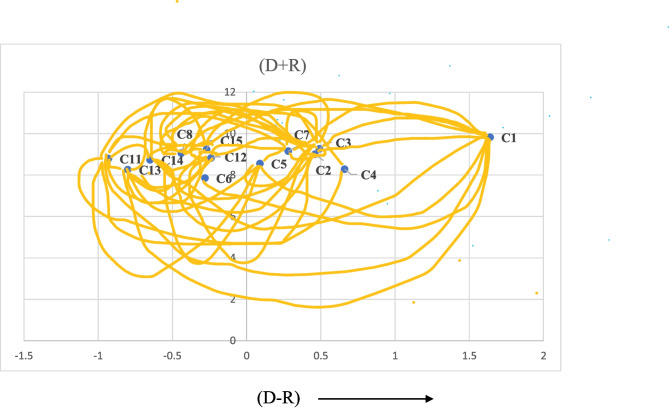
Causal relationship diagram

DEMATEL is used to find the causal relationships among the challenges. The DEMATEL results are shown in Table 7 which has been achieved by using Eqs. (2), (3) and (4). A threshold of α = 0.36 is the significant value obtained by averaging the entries of total relation matrix. The inner dependency matrix is obtained by excluding values lower than the threshold value in Table 7, which can be referred in Table A7 of the supplementary material. This shows the retention of only significant relationships. Based on these relationships, the causal relationship diagram is depicted in Fig. 3. In Table 7, ‘D + R’ values indicate the importance of the criteria which is the degree of the relation of a challenge with the other challenges. A higher value of ‘D + R’ means that the challenge is involved in more relationships with other challenges.

In Table 7, ‘D-R’ values indicate the kind of relation the challenge has with other challenges, whether a cause or effect relationship. The positive values of ‘D-R’ means that the challenge comes under the ‘Cause’ group and influences the other challenges. Negative values of ‘D-R’ indicate that the challenge comes under the ‘Effect’ group and gets influenced by other challenges. A causal relationship diagram is being made by mapping the D + R and D-R values of the respective challenges, as shown in Fig. 3.

According to ‘D-R’ values in the DEMATEL analysis in Table 7, the sub-challenges that support positive values or come in the ‘Cause group’ are Resistance to Change (C1), Lack of Vision and Mission regarding Waste Management (C2), Lack of Strategic Planning for Sustainable Operations (C3), Lack of Top Management Support (C4), Privacy Issues (C5) and High Cost Involved (C7). The sub-challenges that support negative values or come under the ‘Effect group’ are Lack of Trust among Supply Chain Partners (C6), Lack of Financial Support (C8), Lack of Government Policies for BT enabled Waste Management (C11), Lack of Knowledge and Qualified Expertise (C12), Technical Challenges to BT controlled Recyclable Waste Management (C13), Lack of Proper Infrastructure (C14) and Lack of Standards and Regulations regarding Waste Recycling (C15). Figure 3 shows the causal relationships among the sub-challenges, where the sub-challenges with negative values of ‘D-R’ come under the ‘Effect Group’ and the ones with positive values come under the ‘Cause Group’.

## Discussion

In this paper, an effort has been made to explain why BT is appropriate for healthcare waste management and what challenges will arise when the technology is adopted for the management of recyclable hospital waste. BWM is used to rank the challenges to BT adoption in the Indian healthcare sector. Technical and Regulatory Challenges category tops the list followed by Organizational and Management category. Then comes Supply Chain and Collaboration challenges and finally Economic and Environmental Challenges falls last in the prioritization. The rankings obtained for the challenges with the help of BWM are as follows: Lack of Government Policies for BT enabled Waste Management (C11) > Lack of Strategic Planning for Sustainable Operations (C3) > Lack of Knowledge and Qualified Expertise (C12) > Lack of Standards and Regulations regarding Waste Recycling (C15) > High Cost of Technology and Environmentally Sustainable Operations (C7) > Lack of Top Management Support (C4) > Technical Challenges to BT controlled Recyclable Waste Management (C13) > Lack of Financial Support (C8) > Lack of Trust among Supply Chain Partners (C6) > Unclear Vision and Mission regarding Waste Management (C2) > Lack of Proper Infrastructure (C14) > Resistance to Change (C1) > Privacy Issues (C5) > Lack of Environmental Awareness and Green Practices (C9) > Uncertain Return on Investment (C10).

A sensitivity analysis classifies the sub-challenges into high, medium, and low-intensity sub-challenges. High-intensity sub-challenges include Lack of Government Policies for BT enabled Waste Management, Lack of Strategic Planning for Sustainable Operations, Lack of Knowledge and Qualified Expertise, Lack of Standards and Regulations regarding Waste Recycling, High-cost of Technology and Environmentally Sustainable Operations, and Lack of Top Management Support and short-term strategies to tackle them as they deserve immediate attention of managers. Lack of Environmental Awareness and Green Practices and Uncertain Return on Investment falls under low-intensity sub-challenges and can be dealt with long-term strategies. The rest of the sub-challenges fall under medium intensity and need medium-term strategies for confronting.

The two lowest intensity sub-challenges resulting from BWM, and the sensitivity analysis are taken out. DEMATEL analysis is then performed with the remaining thirteen sub-challenges to check their causal relationships. As per the ‘D-R’ values in the DEMATEL analysis, the top three sub-challenges with the highest D-R values are Resistance to Change (C1), Lack of Top Management Support (C4), and Lack of Strategic Planning for Sustainable Operations (C3). This means that these sub-challenges have the most relationships with other sub-challenges than the rest and highly impact the rest of the system. Therefore, eradication of these sub-challenges shall be the managers' priority for effective adoption of BT for recyclable waste management in the Indian healthcare sector.

People tend to work in the same way which show ‘Resistance to Change (C1)’, a cause factor is due to human nature. In healthcare waste management, confidentiality of information related to waste generated in hospitals as well as their respective disposal sites is essential. It becomes a challenge while applying BT in this domain and maintaining trust among different stakeholders at the same time which can lead to an effect of ‘Lack of Collaboration among Supply Chain Partners (C6)’ (Agbo and Mahmoud 2020). This is also due to a ‘Privacy Issues (C5)’ and unawareness about the technology. Moreover, the information confidentiality in healthcare waste management can make BT application difficult to maintain confidential information about hospital waste and its disposal (Bamakan et al. 2022). People need to be made aware of how the blockchain can be used to manage recycled waste. It is challenging to gain acceptance at the grassroots level without knowing its benefits. ‘Lack of Top Management Support (C4)’, one of the main cause factors in DEMATEL analysis, plays a crucial role in introducing new strategies in healthcare organizations. It motivates the development of a learning environment and builds the confidence of its members to reduce their resistance to change. To have an efficient waste management system, active participation of both top management as well as the healthcare stakeholders is must.

‘Unclear Vision and Mission regarding Waste Management (C2)’ and ‘Lack of Strategic Planning for Sustainable Operations (C3)’ are the other causal factors that managers need to give attention. The vision and mission of the top management are essential for BT adoption to manage issue of recyclable healthcare waste. This leads to an effect of ‘Lack of Proper Infrastructure (C14)’. Batubara et al. (2018) suggested that there is a need for a better infrastructure in the Indian healthcare environment for the adoption of BT. It is essential to deal with the large volume of healthcare data generated everyday with optimum cost and time involved. Healthcare organizations need to set clear goals and use the technology adequately to measure and track progress. Clear vision and subsequent mission are essential for linking the organizational objectives with BT and RL implementation (Govindan et al. 2022). Government advertisements and campaigning can help promote the use of technology for sustainable RL practices. Introduction of suitable government policies can help reduce the gap between environmental awareness and environmental practice. This can help improve the stakeholders’ support, both from the government and the management side.

‘Unclear Vision and Mission’, ‘Lack of Strategic Planning’ and ‘High Cost Involved (C7)’ also leads to effects of ‘Lack of Financial Support (C8)’, ‘Lack of Government Policies for BT enabled Waste Management’, ‘Lack of Knowledge and Qualified Expertise (C12)’, ‘Technical Challenges to BT controlled Recyclable Waste Management (C13)’ and ‘Lack of Standards and Regulations regarding Waste Recycling (C15)’. The ambiguity in the rules and regulations associated with the adoption of BT also act as a hindrance for its use in the healthcare waste management (Bamakan et al 2022).

Waste management regulations for Indian municipal corporations lacks alignment with BT. The integration of BT enabled infrastructure in recyclable waste management can provide a digital platform for efficient monitoring and working of the system. Policy making is essential for recyclable waste management problem and its conversion into a profitable business model. The newly developed waste management policies should be in line with BT principles. Regulatory policies are essential as waste management requires strict policies for making changes in the present system. Stakeholders attitude towards waste problems also depends on the formulated policies. Targeted government policies can make people aware about blockchain and its use for changing the traditional way of handling recyclable waste. Further, public–private partnerships can help in lack of funds and subsidy for waste management. The findings of Lu (2019) further supported that different users use different operating systems as well as software, while operating the technology, which leads to the technical issue of interoperability. As a result, different blockchains are unable to connect with others (Savelyev 2018). This can also make BT application in healthcare waste management more time consuming (Bamakan et al. 2022). The Indian Government shall provide suitable standards and regulations for the use of BT. The ambiguities in the rules and regulations makes BT use in healthcare waste management a difficult task. Policies shall also be framed to avoid spread of infections by specifying segregation, collection, storage, transportation, and treatment of recyclable waste. This will make different blockchains interoperable (Douladiris et al. 2020). Feng et al. (2020) supported the result that correct sharing of information among the desired actors is essential that will help to reduce the ‘Lack of Trust among the stakeholders. The design constraints associated with the technology can also hinder the accessing of suitable healthcare waste management information.

BT has the potential to restructure the Indian RL system and improve status of healthcare waste management with its unique advantages (Bamakan et al. 2022). The technology can be used for waste identification, its collection, separation and for managing the hospital sewage system (Bamakan et al. 2022). BT applications can help in better identification of generated hospital waste, its tracking and its segregation into hazardous or recyclable waste, which can be further dealt with RL. This would facilitate proper waste disposal and reduce traffic load around the hospital premises. The records of the generated waste on blockchain can help to decide how it can be reused or recycled in future. It can help identify the healthcare organizations that follow sustainable waste management practices. Suitably, incentivization can be done for following waste management practices and fines can be imposed on the ones that are non-compliant with the laws (Govindan et al. 2022). Proper management and eradication of the challenges are essential for reducing the losses and improving the Indian healthcare system. Due to limited time and finance constraints, the healthcare organizations shall first focus on the critical or top-ranked challenges for the timely adoption of BT in the Indian healthcare sector.

BT cannot be compared with other emerging technologies as it is still in the early stages of adoption in the Indian healthcare sector. Therefore, BT cannot replace any existing technology, and the difficulties in implementing it for the management of medical waste are unique. Even the sub-challenges like Unclear Vision and Mission regarding Waste Management (C2), Lack of Environmental Awareness and Green Practices (C9), Lack of government policies for BT enabled waste management (C11), technical challenges to BT controlled recyclable waste management (C13) are technology specific. Indeed, the goals for using a particular technology for waste management, its effects on the environment, governmental regulations, and technical difficulties can vary.

## Theoretical and managerial implications

### Theoretical implications

This study has made a unique contribution by identifying and offering a thorough understanding of the challenges for BT adoption to manage RL issue of recyclable healthcare waste. The adoption of BT-based services for handling RL depends on Organizational and Management Challenges, Supply Chain and Collaboration Challenges, Economic and Environmental Challenges, and Technological and Regulatory Challenges. Apart from novel addition to the previously published literature, this work helps researchers and academicians explore BT's role in RL management and understand its potential in restructuring of logistics system for better operations. This work tries to build a perspective regarding BT’s use in Indian healthcare waste management and what hindrances need to be removed for achieving it. This article is helpful particularly for the Indian healthcare industry as it explains the cause-and-effect relationships among the challenges. It will assist the top management of healthcare organizations to identify the dominant challenges that need to be removed at an initial stage. The hybrid approach of BWM and DEMATEL used in this study will help researchers and practitioners to assess the challenges’ weights and analyse their cause-and-effect relationships. This study further helps to understand different measures that can help reduce the intensity of different barriers hampering BT’s adoption in the Indian healthcare sector to manage recyclable waste. This hybrid approach has helped provide a robust solution to solve this research problem.

### Managerial implications

Healthcare decision-makers must take action to advance knowledge of the blockchain and how it works in the area of healthcare waste management and raise environmental awareness among stakeholders. BT application can be extended to different aspects of healthcare waste management. The BT application can be expanded to cover various facets of waste management in the medical industry. Waste can be identified based on various characteristics, such as toxicity, substance, etc., and the associated data can be stored on a blockchain platform that has been specially designed (Liu et al. 2021). Information exchange between industries that use hospital waste and industries that produce waste can be facilitated by the use of BT in hospital waste classification. (Gopalakrishnan et al. 2021). It will aid in increasing productivity and encourage appropriate waste disposal (Sløgedal and Starling 2020). Both the environment and human health may be impacted by hospital waste. One method of using BT that could result in effective tracking and minimize its negative effects is waste encryption. BT can assist with both on-site and off-site waste transportation. It may make proper waste exchange within hospitals easier (Pournader et al. 2020). In order to increase transportation transparency, BT is able to track hospital waste vehicles outside of the hospital setting. The route can be clarified by recording the transportation path outside of the hospitals. Data recording, transparency, review, and acceleration of information exchange related to hospital sewage system are all made possible with the effective use of BT in healthcare waste management processes (Dogo et al. 2019). By using BT to record information about sewage disposal systems, potential flaws can be found and repaired, lowering costs and environmental pollution. Therefore, top management as well as the government need to inform the stakeholders about the BT applications in healthcare waste management. This step will help managers tackle the ‘Lack of Vision and Mission regarding waste management (C2)’, ‘Lack of strategic planning for sustainable operations (C3)’ and ‘Lack of top management support (C4)’, the three effect group sub-challenges.

Periodic training sessions of the healthcare workforce can help overcome the ‘Resistance to change (C1)’, a cause group sub-challenge and fear of adapting to a new system. Suitable organizational training in consultation with the healthcare stakeholders is needed to overcome the effect group sub-challenge of ‘Lack of knowledge and qualified expertise (C12)’. Lack of trust and collaboration among supply chain partners has become common during the COVID scenario. It will be easy to integrate multiple healthcare stakeholders through BT implementation. BT adoption for RL operations in healthcare will help managers to build more resilient healthcare supply chains with multiple healthcare stakeholders connected. The application of BT can help reduce hospital’s falsification of environmental data, reducing environmental pollution and can become an answer to the effect group sub-challenge of ‘Lack of trust among supply chain partners (C6)’. The Indian Government shall propose rewards and benefits for the technical expertise of BT to stop their migration to other countries for better earnings due to the present ‘Lack of government policies for BT enabled waste management (C11)’. Colossal funding is required to set up the blockchain infrastructure in the healthcare domain to mitigate ‘High cost of technology and environmentally sustainable operations (C7)’. The Indian Government and the top management of hospitals must think of their contribution to financial support and infrastructure development for the technology in post COVID-19 era to tackle effect group sub-challenges of ‘Lack of financial support (C8)’ and ‘Lack of proper infrastructure (C14)’. To fully capitalise the potential of BT, the authorities need to invest in digital infrastructure as well as the technical staff. Government’s regulatory structure and suitable policies are necessary for India as different blockchain based healthcare start-ups follow different protocols. The policymakers need to think about how the technology can be utilised to interconnect private, public, and non-profit organizations in the healthcare sector during this COVID phase. Big companies avoid investing in this sector due to the policy issues. So, healthcare policymakers shall work at a supranational level to disrupt BT's healthcare services. These steps are essential for managers to mitigate ‘Lack of standards and regulations regarding waste recycling (C15), ‘Privacy Issues (C5)’ and ‘Technical Challenges to BT controlled recyclable waste management (C13)’. Mitigation of the mentioned challenges effectively needs stakeholder’s involvement. Therefore, the Indian Government and policymakers shall consider stakeholder’s views before drafting the required policies for BT adoption for managing RL issues related to healthcare waste.

## Conclusion, limitations and future scope

The healthcare industry plays an essential role in boosting a nation’s economy and progress. In India, it has significantly impacted the GDP, and its efficient working is necessary for maintaining the nation’s development. Adoption of technology like blockchain, especially hybrid blockchains, can significantly improve the efficiency of healthcare working for managing recyclable waste. In this study, we identified and classified fifteen challenges to BT adoption in the Indian healthcare waste management scenario into four categories with the help of the literature and experts’ views. A combined methodology of BWM-DEMATEL has been used in this work to analyse the challenges. According to BWM, Technological and Regulatory challenges are the top-ranked, followed by Organizational and Management challenges, Supply Chain and Collaboration challenges, and Economic and Environmental challenges. Lack of Government Support and Policies (C11), Lack of Strategic Planning (C3), and Lack of Knowledge and Qualified Expertise (C12) are the top three dominant sub-challenges. Lack of Standards and Regulations (C15), Lack of Top Management Support (C4), and High Cost Involved (C7) are the other high-intensity sub-challenges that ask for more consideration from managers. The division of challenges into high, medium, and low intensity has been justified with the help of sensitivity analysis. The DEMATEL analysis further identified the challenges’ cause-and-effect contextual relationships. Resistance to Change (C1), Lack of Vision and Mission regarding Waste Management (C2), Lack of Strategic Planning for Sustainable Operations (C3), Lack of Top Management Support (C4), Privacy Issues (C5) and High Cost Involved (C7) are the sub-challenges that come under cause group, driving the other effect group sub-challenges, deserve immediate attention.

The results indicated that the Indian healthcare waste management system is not yet prepared to implement BT widely. Prior to making any strategic decisions for removing the barriers to its implementation, the policymakers and the various stakeholders must create a roadmap. First, necessary adjustments must be made to organization structure and policies. Both the government and top management in the healthcare industry need to make strategic investments in digital infrastructure and the development of a technically skilled workforce. For the widespread use of BT for healthcare waste management, a global legal and regulatory framework is ideal. The managers should first concentrate on eliminating the challenges that drive the effect group, or the causal group. In addition to making BT adoption easier, removing these obstacles will encourage more digital innovations.

This study has few limitations. Since the results are based on the opinion of experts, they could differ with a change of experts. Therefore, further consolidation of the results can be tackled as future work. Moreover, a limited number of experts’ views were taken due to less availability of BT experts in the Indian healthcare context. Future studies should involve higher number of experts for results’ validation. The integration of BT with other technologies such as cloud computing, fog computing and IoT in future would result in better possibilities for managing healthcare waste. Future work can include BT’s potential to meet sustainability requirements in hospital waste management. This study is done in the Indian context and can be helpful for other emerging economies. A cross-sector comparative analysis can be performed as future work. Future studies can include more stakeholders’ opinions on the challenges in Indian healthcare supply chains.

## Supplementary Information

Below is the link to the electronic supplementary material.

Supplementary file1 (DOCX 50 KB)

## Data Availability

The data that support the findings of this study are available in the supplementary file and further details are available from the corresponding author on request.

## Notations

BT: Blockchain Technology
RL: Reverse Logistics
BWM: Best Worst Method
DEMATEL: Decision Making Trial and Evaluation Laboratory
MCDM: Multi-Criteria Decision Making
COVID-19: Coronavirus Disease-2019
PPE: Personal Protective Equipment
ISM: Interpretive Structural Modelling
MICMAC: Cross- Impact Matrix Multiplication applied to Classification
EDAS: Evaluation based on Distance from Average Solution
TOPSIS: Technique for Order of Preference by Similarity to Ideal Solution
ANP: Analytic Network Process
AHP: Analytic Hierarchy Process
