# Supplementary material for: Blockchain adoption challenges in the healthcare sector: a waste management perspective
Source: Oper Manag Res. 2023 Sep 25;18(2):518–36. doi: 10.1007/s12063-023-00413-9 (PMC12231271; doi:10.1007/s12063-023-00413-9)
Supplement: Supplementary file 1 — Supplementary file1 (DOCX 50 KB) [file 12063_2023_413_MOESM1_ESM.docx]

**Supplementary Material**

Table A1: ‘Best-to-Others’ and ‘Others-to-Worst’ Matrix for (CR=0.04)

| **Best-to-Others (BO)** | **Lack of Trust among supply chain partners** | **Privacy Issues** | **High Cost of technology and environmentally sustainable operations** |
| --- | --- | --- | --- |
| High Cost Involved | 3 | 2 | 1 |
| **Others-to-Worst (OW)** | | **Worst Challenge (Lack of Trust)** | |
| Lack of Trust among Supply Chain Partners | | 1 | |
| Privacy Issues | | 2 | |
| High Cost of technology and environmentally sustainable operations | | 3 | |

Table A2: ‘Best-to-Others’ and ‘Others-to-Worst’ Matrix for (CR=0.05)

| **Best-to-Others (BO)** | **Lack of Government Policies for BT enabled waste management** | **Lack of Knowledge and Expertise** | **Technical Challenges to BT controlled recyclable waste management** | **Lack of Infrastructure** | **Lack of Standards and Regulations regarding waste recycling** |
| --- | --- | --- | --- | --- | --- |
| Lack of Government Policies for BT enabled Waste Management | 1 | 2 | 4 | 5 | 3 |
| **Others-to-Worst (OW)** | | | **Worst Challenge (Lack of Infrastructure)** | | |
| Lack of Government Policies for BT enabled Waste Management | | | 5 | | |
| Lack of Knowledge and Expertise | | | 4 | | |
| Technical Challenges to BT controlled Recyclable Waste Management | | | 2 | | |
| Lack of Proper Infrastructure | | | 1 | | |
| Lack of Standards and Regulations regarding Waste Recycling | | | 3 | | |

Table A3: ‘Best-to-Others’ and ‘Others-to-Worst’ Matrix for (CR=0.1)

| **Best-to-Others (BO)** | **Lack of Financial Support** | **Lack of Environmental Awareness and Green Practices** | **Uncertain ROI** |
| --- | --- | --- | --- |
| Lack of Financial Support | 1 | 3 | 4 |
| **Others-to-Worst (OW)** | | **Worst Challenge (Uncertain ROI)** | |
| Lack of Financial Support | | 4 | |
| Lack of Environmental Awareness and Green Practices | | 3 | |
| Uncertain Return on Investment | | 1 | |

Table A4: ‘Best-to-Others’ and ‘Others-to-Worst’ Matrix for (CR= 0.05)

| **Best-to-Others (BO)** | **Resistance to Change** | **Lack of Mission and Vision regarding waste management** | **Lack of Strategic Planning for sustainable operations** | **Lack of Top Management Support** |
| --- | --- | --- | --- | --- |
| Lack of Strategic Planning for Sustainable Operations | 5 | 4 | 1 | 2 |
| **Others-to-Worst (OW)** | | **Worst Challenge (Resistance to Change)** | | |
| Resistance to Change | | 1 | | |
| Lack of Mission and Vision regarding waste management | | 2 | | |
| Lack of Strategic Planning for Sustainable Operations | | 5 | | |
| Lack of Top Management Support | | 3 | | |

Table A5: Variation in Weights of Challenges with Sensitivity Analysis

| **Challenges** | **Normal Weights (0.51)** | **Preference Weights for Challenges** | | | | | | | | |
| --- | --- | --- | --- | --- | --- | --- | --- | --- | --- | --- |
|  |  | **0.1** | **0.2** | **0.3** | **0.4** | **0.5** | **0.6** | **0.7** | **0.8** | **0.9** |
| **C1** | 0.0243 | 0.0450 | 0.0396 | 0.0342 | 0.0288 | 0.0234 | 0.0180 | 0.0126 | 0.0072 | 0.0018 |
| **C2** | 0.0378 | 0.0700 | 0.0616 | 0.0532 | 0.0488 | 0.0364 | 0.0280 | 0.0196 | 0.0112 | 0.0028 |
| **C3** | 0.1350 | 0.2500 | 0.2200 | 0.1900 | 0.1600 | 0.1300 | 0.1000 | 0.0700 | 0.0400 | 0.0100 |
| **C4** | 0.0729 | 0.1350 | 0.1188 | 0.1026 | 0.0864 | 0.0702 | 0.0540 | 0.0378 | 0.0216 | 0.0054 |
| **C5** | 0.0238 | 0.0442 | 0.0391 | 0.0340 | 0.0289 | 0.0238 | 0.0187 | 0.0136 | 0.0085 | 0.0034 |
| **C6** | 0.0406 | 0.0754 | 0.0667 | 0.0580 | 0.0493 | 0.0406 | 0.0319 | 0.0232 | 0.0145 | 0.0058 |
| **C7** | 0.0756 | 0.1404 | 0.1242 | 0.1080 | 0.0918 | 0.0756 | 0.0594 | 0.0432 | 0.0270 | 0.0108 |
| **C8** | 0.0496 | 0.0868 | 0.0806 | 0.0744 | 0.0682 | 0.0620 | 0.0558 | 0.0496 | 0.0434 | 0.0372 |
| **C9** | 0.0200 | 0.0350 | 0.0325 | 0.0300 | 0.0275 | 0.0250 | 0.0225 | 0.0200 | 0.0175 | 0.0150 |
| **C10** | 0.0104 | 0.0182 | 0.0169 | 0.0156 | 0.0143 | 0.0130 | 0.0117 | 0.0104 | 0.0091 | 0.0078 |
| **C11** | 0.2091 | 0.0410 | 0.0820 | 0.1230 | 0.1640 | 0.2050 | 0.2460 | 0.2870 | 0.3280 | 0.3690 |
| **C12** | 0.1224 | 0.0240 | 0.0480 | 0.0720 | 0.0960 | 0.1200 | 0.1440 | 0.1680 | 0.1920 | 0.2160 |
| **C13** | 0.0612 | 0.0120 | 0.0240 | 0.0360 | 0.0480 | 0.0600 | 0.0720 | 0.0840 | 0.0960 | 0.1080 |
| **C14** | 0.0357 | 0.0070 | 0.0140 | 0.0210 | 0.0280 | 0.0350 | 0.0420 | 0.0490 | 0.0560 | 0.0630 |
| **C15** | 0.0816 | 0.0160 | 0.0320 | 0.0480 | 0.0640 | 0.0800 | 0.0960 | 0.1120 | 0.1280 | 0.1440 |
| **Total** | 1 | 1 | 1 | 1 | 1 | 1 | 1 | 1 | 1 | 1 |

Table A6: Generating the Direct-Relation Matrix

|  | **C1** | **C2** | **C3** | **C4** | **C5** | **C6** | **C7** | **C8** | **C11** | **C12** | **C13** | **C14** | **C15** | **sum** |
| --- | --- | --- | --- | --- | --- | --- | --- | --- | --- | --- | --- | --- | --- | --- |
| **C1** | 0.00 | 3.00 | 3.33 | 1.67 | 2.33 | 2.33 | 2.67 | 3.00 | 2.67 | 2.33 | 2.00 | 2.67 | 3.67 | 31.67 |
| **C2** | 2.33 | 0.00 | 2.00 | 1.33 | 2.00 | 2.00 | 2.00 | 2.67 | 2.67 | 1.67 | 2.67 | 2.67 | 3.00 | 27.00 |
| **C3** | 2.33 | 2.33 | 0.00 | 2.00 | 1.67 | 1.33 | 3.33 | 3.00 | 2.67 | 1.33 | 2.00 | 3.33 | 3.00 | 28.33 |
| **C4** | 2.00 | 2.00 | 2.33 | 0.00 | 1.00 | 2.33 | 1.00 | 2.67 | 3.00 | 1.67 | 2.00 | 1.67 | 1.33 | 23.00 |
| **C5** | 2.33 | 2.33 | 1.00 | 2.00 | 0.00 | 2.33 | 1.67 | 2.00 | 2.33 | 1.67 | 3.00 | 2.00 | 2.00 | 24.67 |
| **C6** | 2.67 | 1.33 | 1.33 | 2.33 | 1.67 | 0.00 | 1.33 | 2.00 | 1.33 | 1.67 | 1.67 | 1.00 | 2.00 | 20.33 |
| **C7** | 1.67 | 2.67 | 2.33 | 1.67 | 2.00 | 2.00 | 0.00 | 1.67 | 3.00 | 3.33 | 2.33 | 2.33 | 3.00 | 28.00 |
| **C8** | 2.00 | 1.33 | 2.67 | 1.33 | 2.33 | 1.67 | 2.00 | 0.00 | 3.33 | 2.67 | 2.67 | 2.33 | 3.00 | 27.33 |
| **C11** | 1.33 | 1.00 | 2.00 | 2.00 | 2.33 | 1.33 | 2.67 | 3.33 | 0.00 | 3.00 | 3.00 | 2.33 | 1.67 | 26.00 |
| **C12** | 1.33 | 1.33 | 2.00 | 1.33 | 2.00 | 1.67 | 3.33 | 2.67 | 2.67 | 0.00 | 2.00 | 3.00 | 2.67 | 26.00 |
| **C13** | 1.00 | 2.67 | 1.00 | 1.00 | 3.00 | 1.67 | 2.00 | 2.33 | 3.00 | 2.00 | 0.00 | 2.33 | 1.00 | 23.00 |
| **C14** | 2.00 | 2.00 | 2.33 | 1.67 | 2.00 | 1.33 | 1.33 | 2.00 | 2.67 | 2.33 | 2.00 | 0.00 | 3.67 | 25.33 |
| **C15** | 1.67 | 2.67 | 3.33 | 1.00 | 1.33 | 2.33 | 2.67 | 2.67 | 2.00 | 3.33 | 2.00 | 3.33 | 0.00 | 28.33 |

Maximum = 0.03

Table A7: Inner Dependence Matrix

|  | **C1** | **C2** | **C3** | **C4** | **C5** | **C6** | **C7** | **C8** | **C11** | **C12** | **C13** | **C14** | **C15** |
| --- | --- | --- | --- | --- | --- | --- | --- | --- | --- | --- | --- | --- | --- |
| **C1** |  | 0.43 | 0.46 |  | 0.41 | 0.38 | 0.45 | 0.51 | 0.52 | 0.46 | 0.45 | 0.49 | 0.53 |
| **C2** |  |  | 0.37 |  |  |  | 0.38 | 0.44 | 0.45 | 0.38 | 0.41 | 0.43 | 0.45 |
| **C3** | 0.36 | 0.38 |  |  | 0.36 |  | 0.43 | 0.47 | 0.47 | 0.39 | 0.41 | 0.47 | 0.47 |
| **C4** |  |  |  |  |  |  |  | 0.39 | 0.41 |  |  |  |  |
| **C5** |  |  |  |  |  |  |  | 0.39 | 0.41 |  | 0.39 | 0.38 | 0.39 |
| **C6** |  |  |  |  |  |  |  |  |  |  |  |  |  |
| **C7** |  | 0.38 | 0.39 |  | 0.36 |  |  | 0.42 | 0.47 | 0.44 | 0.41 | 0.43 | 0.46 |
| **C8** |  |  | 0.39 |  | 0.36 |  | 0.38 | 0.36 | 0.48 | 0.42 | 0.41 | 0.43 | 0.45 |
| **C11** |  |  | 0.36 |  |  |  | 0.38 | 0.44 | 0.36 | 0.41 | 0.41 | 0.41 | 0.39 |
| **C12** |  |  | 0.36 |  |  |  | 0.40 | 0.42 | 0.44 |  | 0.38 | 0.43 | 0.43 |
| **C13** |  |  |  |  |  |  |  | 0.37 | 0.41 |  |  | 0.37 |  |
| **C14** |  |  | 0.37 |  |  |  |  | 0.40 | 0.43 | 0.38 | 0.37 |  | 0.45 |
| **C15** |  | 0.39 | 0.42 |  |  |  | 0.41 | 0.45 | 0.45 | 0.44 | 0.41 | 0.47 | 0.38 |
| **sum of column R** | 4.09 | 4.45 | 4.68 | 3.53 | 4.36 | 4.04 | 4.78 | 5.40 | 5.63 | 4.98 | 4.97 | 5.29 | 5.42 |
|  |  |  |  |  |  |  |  |  |  |  | **α** | **0.36** |  |

Table A8: Demographic Profile

| **Professional from Healthcare Units** | **Educational Background** | **Experience** | **Approached** | **Responded** |
| --- | --- | --- | --- | --- |
| Managers/ CEOs | MBA/ PHD | 4-10 years | 10 | 8 |
| Medical experts and the healthcare industry workers  (e.g., Doctors, physicians, technology expertise, engineers etc.) | MBBS/MD/ B.Tech. | 10-20 years | 9 | 7 |
| **Total** | | | **19** | **15** |

Table A9: Variation in weight of category (Ⅳ) from 0.1 to 0.9

| **Selected Category** | **Normal (0.51)** | **Preference Weights for Various Categories** | | | | | | | | |
| --- | --- | --- | --- | --- | --- | --- | --- | --- | --- | --- |
|  |  | **0.1** | **0.2** | **0.3** | **0.4** | **0.5** | **0.6** | **0.7** | **0.8** | **0.9** |
| **Ⅰ** | 0.27 | 0.50 | 0.44 | 0.38 | 0.32 | 0.26 | 0.20 | 0.14 | 0.08 | 0.02 |
| **Ⅱ** | 0.14 | 0.26 | 0.23 | 0.20 | 0.17 | 0.14 | 0.11 | 0.08 | 0.05 | 0.02 |
| **Ⅲ** | 0.08 | 0.14 | 0.13 | 0.12 | 0.11 | 0.10 | 0.09 | 0.08 | 0.07 | 0.06 |
| **Ⅳ** | 0.51 | 0.10 | 0.20 | 0.30 | 0.40 | 0.50 | 0.60 | 0.70 | 0.80 | 0.90 |
| **Total** | 1 | 1 | 1 | 1 | 1 | 1 | 1 | 1 | 1 | 1 |
